# Non invasive imaging assessment of the biodistribution of GSK2849330, an ADCC and CDC optimized anti HER3 mAb, and its role in tumor macrophage recruitment in human tumor-bearing mice

**DOI:** 10.1371/journal.pone.0176075

**Published:** 2017-04-27

**Authors:** Hasan Alsaid, Tinamarie Skedzielewski, Mary V. Rambo, Kristen Hunsinger, Bao Hoang, William Fieles, Edward R. Long, James Tunstead, Danielle J. Vugts, Matthew Cleveland, Neil Clarke, Christopher Matheny, Beat M. Jucker

**Affiliations:** 1 Bioimaging, Platform Technology & Science, GlaxoSmithKline, King of Prussia, Pennsylvania, United States of America; 2 Target Sciences Target & Pathway, GlaxoSmithKline, King of Prussia, Pennsylvania, United States of America; 3 Integrated Biological Platform Sciences, Platform Technology & Science, GlaxoSmithKline, King of Prussia, Pennsylvania, United States of America; 4 Department of Radiology and Nuclear Medicine, VU University Medical Center, Amsterdam, The Netherlands; 5 Bioimaging, Platform Technology & Science, GlaxoSmithKline, Stevenage, United Kingdom; 6 Biopharm Molecular Discovery, GlaxoSmithKline, Stevenage, United Kingdom; 7 Immunoginicity and Biomarkers, Platform Technology & Science, GlaxoSmithKline, King of Prussia, Pennsylvania, United States of America; Genentech Inc, UNITED STATES

## Abstract

The purpose of this work was to use various molecular imaging techniques to non-invasively assess GSK2849330 (anti HER3 ADCC and CDC enhanced ‘AccretaMab’ monoclonal antibody) pharmacokinetics and pharmacodynamics in human xenograft tumor-bearing mice. Immuno-PET biodistribution imaging of radiolabeled ^89^Zr-GSK2849330 was assessed in mice with HER3 negative (MIA-PaCa-2) and positive (CHL-1) human xenograft tumors. Dose dependency of GSK2849330 disposition was assessed using varying doses of unlabeled GSK2849330 co-injected with ^89^Zr-GSK2849330. In-vivo NIRF optical imaging and ex-vivo confocal microscopy were used to assess the biodistribution of GSK2849330 and the HER3 receptor occupancy in HER3 positive xenograft tumors (BxPC3, and CHL-1). Ferumoxytol (USPIO) contrast-enhanced MRI was used to investigate the effects of GSK2849330 on tumor macrophage content in CHL-1 xenograft bearing mice. Immuno-PET imaging was used to monitor the whole body drug biodistribution and CHL-1 xenograft tumor uptake up to 144 hours post injection of ^89^Zr-GSK2849330. Both hepatic and tumor uptake were dose dependent and saturable. The optical imaging data in the BxPC3 xenograft tumor confirmed the tumor dose response finding in the Immuno-PET study. Confocal microscopy showed a distinguished cytoplasmic punctate staining pattern within individual CHL-1 cells. GSK2849330 inhibited tumor growth and this was associated with a significant decrease in MRI signal to noise ratio after USPIO injection and with a significant increase in tumor macrophages as confirmed by a quantitative immunohistochemistry analysis. By providing both dose response and time course data from both ^89^Zr and fluorescently labeled GSK2849330, complementary imaging studies were used to characterize GSK2849330 biodistribution and tumor uptake in vivo. Ferumoxytol-enhanced MRI was used to monitor aspects of the immune system response to GSK2849330. Together these approaches potentially provide clinically translatable, non-invasive techniques to support dose optimization, and assess immune activation and anti-tumor responses.

## Introduction

ErbB3/HER3 is a member of the epidermal growth factor receptor family of receptor tyrosine kinases comprising HER1 (EGFR), HER2 (ErbB2), HER3 (ErbB3) and HER4 (ErbB4) which play an important role in the development and progression of cancer[1]. HER3 heterodimerization with HER2 and ligand-driven activation is a key driver of the crucial PI3K/Akt pathway. There is growing evidence that in certain cases, HER3 activation and subsequent PI3K/Akt signalling mediates resistance to EGFR- and HER2-directed therapies. This in turn contributes to the emergence of castrate-resistant prostate cancer, plays a role in resistance to anti-estrogen treatment of ER positive breast cancer, and may play a role in the pathogenesis of melanoma, colon cancer, and ovarian cancer. HER3 is expressed in a broad range of solid tumors where its signaling is important in tumorigenesis and drug resistance[2, 3].

Currently, there are a number of anti-HER3 monoclonal antibodies (mAbs) in clinical development[4–6]. GSK2849330 is an IgG1/IGg3, glyco-engineered, humanized monoclonal antibody (mAb). It has species cross reactivity to human, mouse, rat, and cynomolgus non-human primate HER3. It has been engineered with 3 distinct mechanisms of action: a) disruption of ligand-dependent signaling leading to inhibition of HER3 signaling and function; b) enhanced antibody-dependent cell-mediated cytotoxicity (ADCC) by enhanced FcɣR3a binding of effector cells (e.g., NK cells, macrophages) leading to lysis or phagocytosis of HER3 expressing target cells; c) enhanced complement-dependent cytotoxicity (CDC) by enhanced C1q binding and complement activation[7]. The latter two mechanisms provide an opportunity for differentiation from current HER3-directed mAbs in the clinic based on direct killing of both dividing and non-dividing cells, independent of inhibition of downstream signaling.

Use of novel imaging modalities in preclinical models can provide a means to uniquely address questions related to drug pharmacokinetics and pharmacodynamics in vivo, often non-invasively. When properly designed and executed, these studies can inform pharmacokinetic (PK)-pharmacodynamic (PD) relationships and optimal dosing strategies, and provide preclinical proof-of-principle for techniques which then may be translated to PK or PD readouts in clinical settings[8].

Immuno-positron emission tomography (Immuno-PET) of ^89^Zr-labeled-antibodies has been applied to assess the distribution of the antibody and the target in question in the whole body, and to assess the relationship between dose and receptor-occupancy in the targeted organs[9, 10]. This technique has already been employed both preclinically and clinically as evidence of translation[11–14]. The biodistribution of an antibody from whole body to cellular level can be assessed using optical imaging techniques as well after labeling it with a near infrared fluorescent probe; however there are limitations for clinical translational of optical methods[15]. Ferumoxytol (Feraheme^®^) is an ultrasmall superparamagnetic iron oxide (USPIO) MRI contrast agent that is phagocytosed by macrophages. While the agent has been approved by the Food and Drug Administration (FDA) for intravenous treatment of iron deficiency anemia in adult patients with chronic kidney disease, it has been used to measure tumor-associated macrophages in human tumor-bearing mice[16, 17], and used in clinical MR imaging applications as well[18–20].

In the current study, three complementary techniques, two directly tracking the antibody, and one tracking a PD response marker (macrophage recruitment), were employed to assess the disposition and biologic response in vivo in relevant preclinical models. GSK2849330 was labeled with Zirconium-89 (^89^Zr) or with a fluorescent probe (VivoTag 680) for in-vivo Immuno-PET, and in-vivo/ex-vivo optical imaging was used to investigate the biodistribution of GSK2849330 and to determine the relationship between dose and receptor-occupancy in HER3 negative and positive expressing human tumor-bearing mice. In addition, we investigated the effects of GSK2849330 on tumor macrophage recruitment in HER3 positive expressing human tumor-bearing mice using ferumoxytol-enhanced MRI.

## Methods

### Ethical considerations

All procedures were approved by the Institutional Animal Care and Use Committee of GlaxoSmithKline under IAUC # AUP0500 and were specifically designed to minimize animal discomfort. Mice were housed in pathogen free conditions and handled with aseptic techniques. Mice were anesthetized with continuously inhaled isoflurane (1.5–2%), and CO_2_ was used for euthanasia.

The following clinical abnormalities were in place to euthanize animals prior to the experimental endpoint: 1) If animal showed signs of inability and/or unwillingness to ambulate to reach food or water. 2) If lesions were interfering with eating or drinking. 3) If there was an excessive tumor burden: tumors which exceed a maximum diameter of 1.5 cm or interferes with the animal’s ability to eat, drink, or move normally. 4) If tumor exhibited necrosis, ulceration and/or infection. 5) If tumor was bigger than 2500 mm^3^ in volume for two consecutive measurements.

For euthanasia; the animal was placed into a dedicated chamber. Slow flow rate of CO_2_ was used. The animal remained in the chamber and was observed until all signs of movement or breathing ceased. The animal was removed from the chamber, checked again to confirm respiratory arrest, and verified by lack of response to toe pinch and by lack of corneal reflex and then a secondary physical method of euthanasia such as a thoracotomy or cervical dislocation was performed to ensure the irreversibility of the procedure.

### Cell culture and establishing subcutaneous xenograft tumors

Two HER3-positive expressing cell lines, human CHL-1 melanoma cells and BxPC3 pancreatic adenocarcinoma cells[7], and one HER3-negative expressing cell line, MIA-PaCa-2[21], were obtained from the American Type Culture Collection (ATCC, Manassas, VA). All cell lines were cultured in RPMI medium (RPMI 1640, Life Technologies, Carlsbad, CA) containing 10% fetal bovine serum (FBS, Sigma-Aldrich, St. Louis, MO) and maintained in humidified incubators at 37°C under 5% CO_2_. Single cell suspensions were created in 50% Matrigel (Corning^®^ Matrigel^®^ Basement Membrane Matrix, Phenol Red-Free, *LDEV-Free, 10mL (Product #356237)): 50% PBS (v:v) so that a 100 μL injection would deliver 5 x 10^6^ cells per mouse for tumor implantation.

### Animals and tumor measurement

Female C.B-igh-1/IcrTac-Prkdc<scid> mice (Taconic, Cambridge City, IN) of 8–10 weeks of age were used in these studies. Animals were housed in pathogen free conditions and handled with aseptic techniques.

Following tumor cell implantation on the right flank of the mouse, tumors were allowed to grow to a size of 200–300 mm^3^. Tumor size was measured twice per week, the length and width of each tumor were measured using hand-held calipers and the tumor volume was calculated using the following formula:

$$Volume=\frac{\left(Length\times Width^{2}\right)}{2}$$

Mice were randomly assigned into the required number of treatment groups.

### Synthesis and quality control of conjugated ^89^Zr-GSK2849330

GSK2849330 was reacted with p-SCN-Bn-Deferoxamine in a 1:3 ratio (VU University Medical Center, Amsterdam, The Netherlands, or inviCRO, Boston, MA, USA) and subsequently radiolabeled with Zirconium-89 as described previously[22]. The product was purified by size-exclusion chromatography (PD10, GE Healthcare) and analyzed for radiochemical purity by spin filter (30 kDa cut-off Microcon-30 centrifugal filter Merck-Millipore BV, 4 μl sample was mixed with 96 μL wash buffer (10/90 DMSO/50 mM NaOAc + 200 mM sucrose pH 5.5) and applied to the spin filter. The filter spun down at 14000 rpm for 7 min, and subsequently washed twice with 100 μl wash buffer and spun down at 14000 rpm for 7 min. The amount of radioactivity on the filter and in the supernatant were counted in a gamma-counter (LKB-Wallac, compugamma). The supernatant contained non-mAb bound ^89^Zr and the filter contained ^89^Zr-GSK2849330), SE-HPLC (Jasco HPLC system equipped with a Superdex 200 10/30 GL size exclusion column (GE Healthcare Life sciences) using a mixture of 0.05M sodium phosphate, 0.15 M sodium chloride (pH 6.8), and 0.01M NaN3 as the eluent at a flow rate of 0.5 mL/min. The radioactivity of the eluate was monitored using an inline NaI(Tl) radiodetector (Raytest Sockett)) and sodium dodecylsulfate-PAGE with phosphor imager analyses. In vitro binding characteristics of ^89^Zr-GSK2849330 was determined in a binding assay using HER3 antigen coated plates (0.5 μg/well) cells and a serial dilution of ^89^Zr-GSK2849330 (1 μg/ml down to 15.6 ng/mL, 100 μL).

### In-vivo Immuno-PET

Mice bearing either MIA-PaCa-2 tumors (group1, n = 5) or CHL-1 tumors (group 2, n = 5) were administered 0.5 mg/kg (5 MBq) via intravenous (IV) tail vein injection. To evaluate the HER3 specificity of ^89^Zr-GSK2849330 disposition in CHL-1 tumor-bearing mice, a blocking study (group 3) was performed in which mice (n = 5) received 50 mg/kg of GSK2849330, IV 16 hours before ^89^Zr-GSK2849330 injection (0.5 mg/kg, 5 MBq). PET/CT imaging was performed using a preclinical Inveon PET/CT system (Siemens Medical Solutions USA, Inc) at 24, 48, 72, and 144 hours post injection of ^89^Zr-GSK2849330. For image acquisition, mice were anesthetized with continuously inhaled isoflurane (1.5–2%), and physiological parameters were monitored using a BIOVET animal monitoring system. The CT system was calibrated for the center offset, the Hounsfield Unit, and the Dark and Light calibration. A new 3D PET/CT Co-registration Matrix and a PET Normalization file were acquired. The quantification calibration process was performed using an ^18^F phantom. A CT scan (80 kV, 500 μA, 4 binning factor) was performed in each animal (for co-registration with the PET data and for CT-based PET attenuation scan) followed by a 20 minute PET scan. Coincidence events were acquired into list-mode files, and the resulting sinograms histogrammed into 1 frame and reconstructed using Siemens Inveon Acquisition Workplace (version 1.5.0.28) with an iterative algorithm (2D FBP with Ramp projection filter, matrix size 128×128 with a zoom of 1, leading to a reconstructed voxel size of 0.776X0.776X0.796 mm^3^). The volume and the activity of each ^89^Zr-GSK2849330 dose were measured before injection, and the remaining dose in the syringe was measured to accurately calculate the injected dose. The acquired PET images were decay corrected to the injection time for each single animal.

Image analysis was performed using Inveon Research Workplace 4.2 software (Siemens Medical Solutions USA, Inc), 3D regions of interest (ROIs) for the different organs were defined on the CT image and transferred to the co-registered PET image. Quantitative data were presented as a percentage of injected dose per gram of tissue (% ID/g; mean ± SEM).

### Dose escalation study

A dose escalation biodistribution study with ^89^Zr-GSK2849330 was performed in CHL-1 tumor bearing mice to evaluate dose-dependent tumor uptake of GSK2849330. Five dose groups were included in the study (n = 4/group); each group received 2 IV injections: 1) ^89^Zr-GSK2849330 (0.14 mg/kg) and 2) unlabeled GSK2849330 (0, 0.3, 1, 3, and 10 mg/kg). Ex-vivo biodistribution was performed using Wizard 2480 Gamma Counter (PerkinElmer, Hopkinton, MA), organs were collected at 72 hours post injection, and decay corrected to the injection time. The data is reported as %ID/g and presented as mean±SEM.

### Optical imaging

GSK2849330 was labeled with the VivoTag 680 (PerkinElmer, Inc) using the following conjugation process: at room temperature, 10 μl of stock (100 mg/ml) GSK2849330 was mixed with 500 μl 1xPBS and 50 μl sodium bicarbonate buffer. Lyophilized VivoTag 680 was brought back into solution with 10 μl of dry DMSO. 2 μl of the revitalized VivoTag 680 was added to the GSK2849330 solution and placed on a rocker away from light for two hours to allow for conjugation. The mixture was purified by passing it through a Zeba Spin Desalting Colum (7K MWCO, Thermo Scientific, Rockford, IL, USA) for two minutes at 1,000xg. A small sample of the purified GSK2849330 labeled with VivoTag 680 was placed in a SpectraMax M2 spectrophotometer (Molecular Devices Corporation, CA, USA). Absorption of the conjugate was measured at 280 nm (0.685) and 668 nm (1.0935) to calculate the degree of labeling (moles dye per mol protein) to be 2.14 as described in VivoTag^™^ 680XL labeling kit (PerkinElmer, Inc) using the following equations:

$$\text{Protein concentration}:\;\left(\text{M}\right)\;={\text{ A}}_{280}-\left(0.16{\text{xA}}_{668}\text{ of dye}\right)/\text{ ε }\left(\text{molar extinction coefficient}\right).$$

For antibody, ε is 210,000 M^-1^cm^-1^.

$$\text{Dye concentration }\left(\text{M}\right)\;={\text{ A}}_{668}/\text{ ε }\left(\text{molar extinction coefficient}\right).$$

ε is 210,000 M^-1^cm^-1^ for VivoTag 680XL.

DOL (Moles dye per mole protein) = Dye concentration (M)/Protein concentration (M).

Four groups of mice (age 9–10 weeks) bearing BxPC3 xenograft tumors were used in this experiment (n = 4 per group). Group 1 received 5 mg/kg of the VivoTag 680 labeled GSK2849330 (VivoTag 680-GSK2849330) at 24 hours post injection of a blocking dose (50 mg/kg) of non-labeled GSK2849330. Groups 2, 3, and 4 received 5, 1, and 0.5 mg/kg, respectively, of the VivoTag 680-GSK2849330. Mice were fed with a diet depleted of fluorescent substrates to reduce background signal and improve imaging quality. Optical imaging was performed using an MS FX Pro system (Bruker Inc, Billerica, MA.). The system was calibrated according to manufacturer’s instructions. For image acquisition, mice were anesthetized with continuously inhaled isoflurane (1.5–2%). After a white light image was taken for 5 seconds to assess the animal position, a fluorescent image was acquired using the following parameters: excitation filter: 650 nm, emission filter: 700 nm with f-stop set to 5.6 and an exposure time of 20 seconds. The optical data was analyzed using Bruker molecular imaging software (Bruker Corporation).

### Ex-vivo confocal microscopy

GSK2849330 and an IgG control (Jackson Immuno Research INC, Lot # 115069, Code 099-000-003) monoclonal antibodies were labeled with the VivoTag 680 (Perkin Elmer) as described previously. Mice (age 9–10 weeks) bearing CHL-1 xenograft tumors were IV injected with either VivoTag 680 labeled GSK2849330 or IgG control (5 mg/kg). Tumor samples were collected at 0.5, 1, 3, 6, and 24 hours after injection and micron frozen sections of the tumors were cut and placed onto cold slides. Slides were warmed to room temperature until melted then placed in PBS (Sigma Cat# P3813), pH 7.4, prior to further immunostaining. Sections were incubated in 0.5 ug/ml Alexa 488 labeled goat anti—human IgG (Invitrogen Cat# A11013) in PBS for 1 hour at room temperature, rinsed in PBS and mounted in ProLong Gold with DAPI (Invitrogen Cat# P36931). Sections were qualitatively assessed and representative images acquired using a ZEISS LSM 510 Meta confocal using a 20x, 40x and 63x oil immersion lens, with the 405 laser line for nuclei, 488 for the Alexa 488 tagged anti- human secondary, and the 633 line for the VivoTag 680.

### Magnetic resonance imaging (MRI)

Mice (age 9–10 weeks) bearing CHL-1 tumors were treated for 2 weeks with either vehicle (n = 10) or GSK2849330 (25 mg/kg, IP, 3 times/week, n = 10). Imaging was performed on a 9.4 Tesla vertical MRI (Bruker Biospin GmbH, Germany) using a 30 mm volume coil. MRI was performed pre and 24 hours post IV injection of 1.0 mmol [Fe]/kg ferumoxytol (Feraheme^®^, AMAG Pharmaceuticals) on day 16 post-treatment. Ferumoxytol is a USPIO contrast agent which has been used to identify macrophages by providing a negative MRI contrast following macrophage phagocytosis[23]. For image acquisition, mice were anesthetized with continuously inhaled isoflurane (1.5–2%) and a constant body temperature of 37°C was maintained using a circulating-water heating blanket. Respiration was monitored using a respiratory sensor (SA Instruments, Inc., Stony Brook, NY) placed on the abdomen of the animal. Following a scout image, axial T_1_, T_2_, and T_2_*-weighted (T_2_*-w) images were acquired using the following parameters: FOV = 3.5x3.5 cm, Matrix = 128x128, and slice thickness = 1 mm, T_1_ Fast Spin Echo (TR/TE = 1500/6.5 ms, 4 Echos, NEX = 4), T_2_ Fast Spin Echo (TR/TE = 3000/36 ms, 8 Echos, NEX = 8), and T_2_* Multi Gradient Echo (TR/TE = 300/2.2 ms, flip Angle = 30°, NEX = 4).

MR image analysis was performed using Jim 7.0 software (Xinapse Systems, Essex, UK). Regions of interest (ROIs) that define the tumor were used, and the signal intensity of the ROI per scan was measured and normalized to the background noise. The mean S/N for each scan (multiple slices) was calculated using the following equation:

$$S/N=Avr\;{\left(Mean\;Signal\;\left(ROI\right)/Mean\;\left(Noise\right)\right)}_{n},$$

where n represent the number of slices

### Immunohistochemistry

Following in vivo imaging, mice were euthanized and their tumors were harvested. A median incision was made along each tumor in the rostral cordal plane to obtain two halves. The tumors were immersed fixed in 10% neutral buffered formalin overnight and paraffin embedded. A 5.0 μm mid section was cut from one half belonging to each tumor, deparaffinized in xylene followed by descending washes in alcohols and brought to dH_2_0. Immunohistochemistry for F4/80 (clone CI:A3-1, AbD Serotec) was achieved using the Ventana Discovery XT (Ventana Medical Systems, Tuscon, AZ, USA). Sections were double stained with Prussian blue (Polysciences) for presence of ferric iron. Counterstaining was performed with nuclear fast red. Slides were scanned using the Nanozoomer (Hamamatsu). Jpeg images (40x) were processed in Photoshop 6 to isolate different morphological regions exhibited by the tumors. Metamorph (Molecular Devices Corporation, CA, USA) was used to characterize and count cells stained positive for F4/80, Perls’ and F4/80 –Perls’ double stain. Journals were developed to allow for automated cell counting to be performed on a batch of images. Cells per mm^2^ tissue of both vehicle and drug treated groups were exported to Excel and data is presented as mean±SEM.

### Statistical analysis

Data are presented as mean ± standard error of the mean (SEM). Results were analyzed using GraphPad Prism 5.00 (GraphPad Software, Inc). P-value ≤ 0.05 was considered significant, and was calculated using either Unpaired t-test, One-way ANOVA, or Two-way ANOVA, and Bonferroni’s multiple comparison test was used to adjust for the multiple comparisons.

## Results

### Immuno-PET study

**Fig 1:**
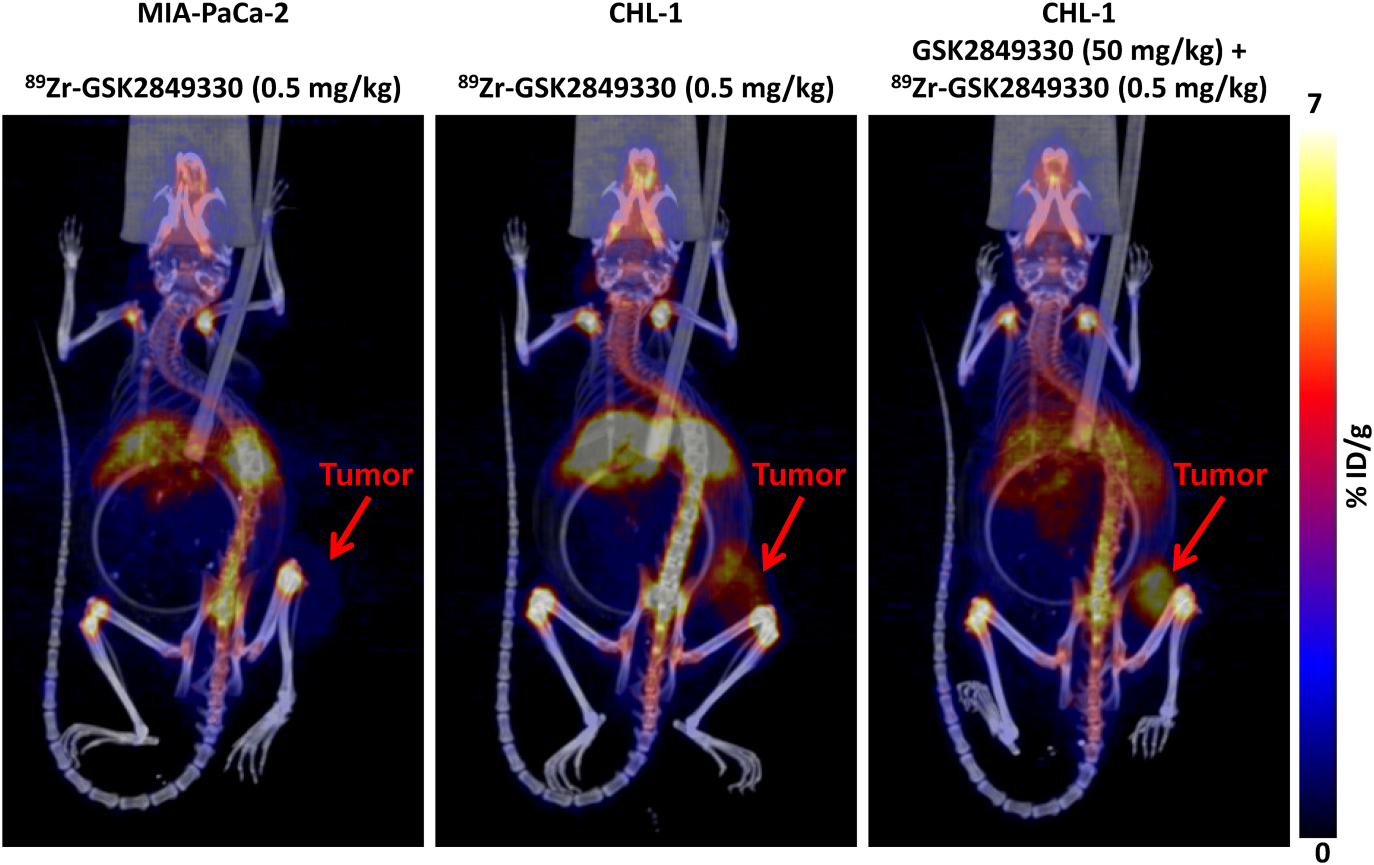
3D co registered PET/CT imaging of MIA-PaCA-2 and CHL-1 xenograft tumor bearing mice at 144 hours post tracer (0.5 mg/kg of ^89^Zr-GSK2849330) injection. The images show high uptake in the CHL-1 tumor (mid-panel) compared to the MIA-PaCa-2 tumor (left-panel). However the uptake was higher in the CHL-1 group (right-panel) which received a non-labeled dose (50 mg/kg) of GSK2849330 16 hours prior to the tracer dose.

The PET/CT images (representative images shown in Fig 1) at 144 hours post injection of ^89^Zr-GSK2849330 (0.5 mg/kg) showed low uptake in HER3-negative MIA-PaCa-2 tumors (group1) compared to HER3-positive CHL-1 tumors (group 2) and CHL-1 tumors in mice pre-treated with a high (50 mg/kg) non-labeled dose of GSK2849330 (group 3).

**Fig 2:**
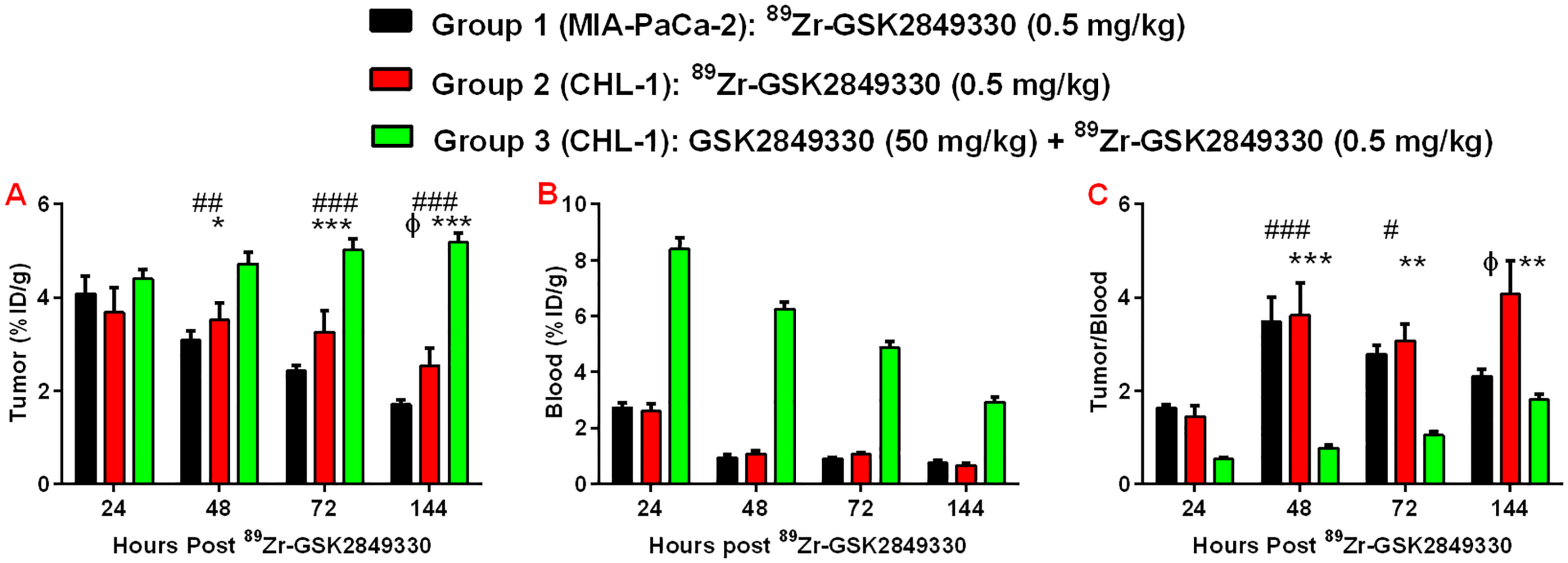
Tumor uptake (A), blood uptake (B) and the normalized tumor to blood ratio (C) at 24, 48, 72, and 144 hours post injection of ^89^Zr-GSK2849330 in MIA-PaCa-2 and CHL-1 xenograft tumor bearing mice as measured in-vivo using Immuno-PET imaging. All groups (1,2, and 3) received 0.5 mg/kg of ^89^Zr-GSK2849330, but group 3 received 50 mg/kg of GSK2849330 16 hours prior to the injection of ^89^Zr-GSK2849330. (*p<0.05, **p<0.01, ***p<0.001, ***p<0.0001: group 2 vs group 3; ^#^p<0.05, ^##^p<0.01, ^###^p<0.001: group 1 vs group 3 using 2-way ANOVA. ^ɸ^p<0.05: group 1 vs group 2 using unpaired t-test). Data is presented as mean±SEM (S1 Table).

Quantitative image analysis of the tumor-associated signals from the PET scans showed significant differences between groups at 144 hours (Fig 2A); lower uptake, expressed as %ID/g, was observed in group 1, with highest uptake in group 3 (group1: 1.7±0.11; group 2: 2.5±0.36; and group 3: 5.18±0.2%ID/g). The ^89^Zr-GSK2849330 uptake in the blood (Fig 2B) was significantly higher in group 3 compared to groups 1 and 2 at all time points, and it decreased significantly over time in group 1 (8.40±0.41 at 24 hours to 2.92±0.19%ID/g at 144 hours, p<0.0001). Of note, the liver was a quantitatively important site of disposition, and significantly lower uptake was observed in the liver for group 3 (5.08±0.29) compared to groups 1 (6.48±0.0.32) and group 2 (7.76±0.77, p<0.01).

The normalized tumor to blood uptake ratio (Fig 2C) showed significantly higher uptake in groups 1 and 2 compared to group 3 at 48h and 72h, and significantly higher uptake in group 2 compared to groups 1 and 3 at 144h post injection. No differences were found between groups 1 and 3 at 144h.

### Dose escalation study

**Fig 3:**
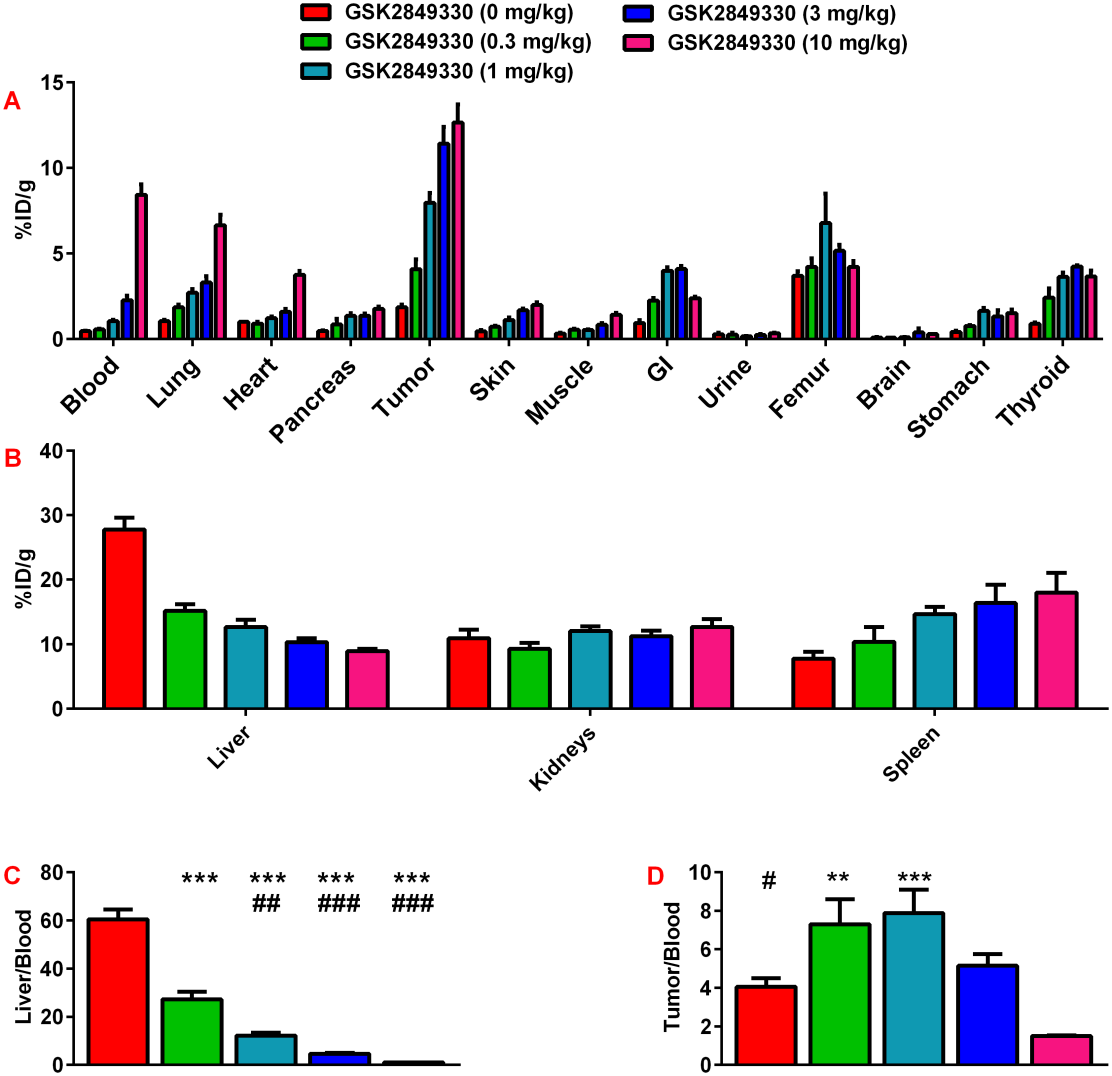
Dose escalation study of ^89^Zr-GSK2849330 in CHL-1 xenograft tumor bearing mice as measured ex-vivo using a Gamma Counter at 72 hours. Each group received a tracer dose (~0.14 mg/kg) of ^89^Zr-GSK2849330 plus a dose of non-labeled GSK2849330 (0, 0.3, 1, 3, or 10 mg/kg) (panels A and B). The normalized liver uptake to blood uptake ratio (Panel C) showed a significant decrease with the increased non labeled GSK2849330 (***p<0.01 vs 0 mg/kg; ^##^p<0.01, ^###^p<0.001 vs 0.3 mg/kg using One-way ANOVA). The normalized tumor uptake to blood uptake ratio (Panel D) showed a significant decrease with the increased non labeled GSK2849330 from 0.3 and 1 mg/kg compared to the 10 mg/kg group (^#^p<0.05 vs 1 mg/kg; ** p<0.01, *** p<0.001 vs 10 mg/kg using One-way ANOVA). Data is presented as mean±SEM (S2 Table).

There was an increase in ^89^Zr-GSK2849330 uptake in CHL-1 xenograft tumors with an increasing non-labeled dose of GSK2849330 (Fig 3A). This increase was associated with increased level of ^89^Zr-GSK2849330 in the blood which may contribute to the increased uptake observed in some highly perfused organs. Notably, the ^89^Zr-GSK2849330 uptake in liver (Fig 3B) was decreased with the increased non-labeled dose of GSK2849330.

The normalized liver to blood ratio confirmed the significant decrease of the ^89^Zr-GSK2849330 uptake in the liver (Fig 3C). However, the normalized tumor to blood ratio (Fig 3D) showed a significant increase from 4.07±0.4 for 0 mg/kg of GSK2849330 to reach a plateau of 7.31±1.3, and 7.89±0.6 for 0.3, and 1 mg/kg of GSK2849330, respectively. This ratio decreased significantly with the higher non-labeled doses of GSK2849330 to 5.15±0.6 and 1.5±0.04 for 3 and 10 mg/kg, respectively.

### Optical imaging

**Fig 4:**
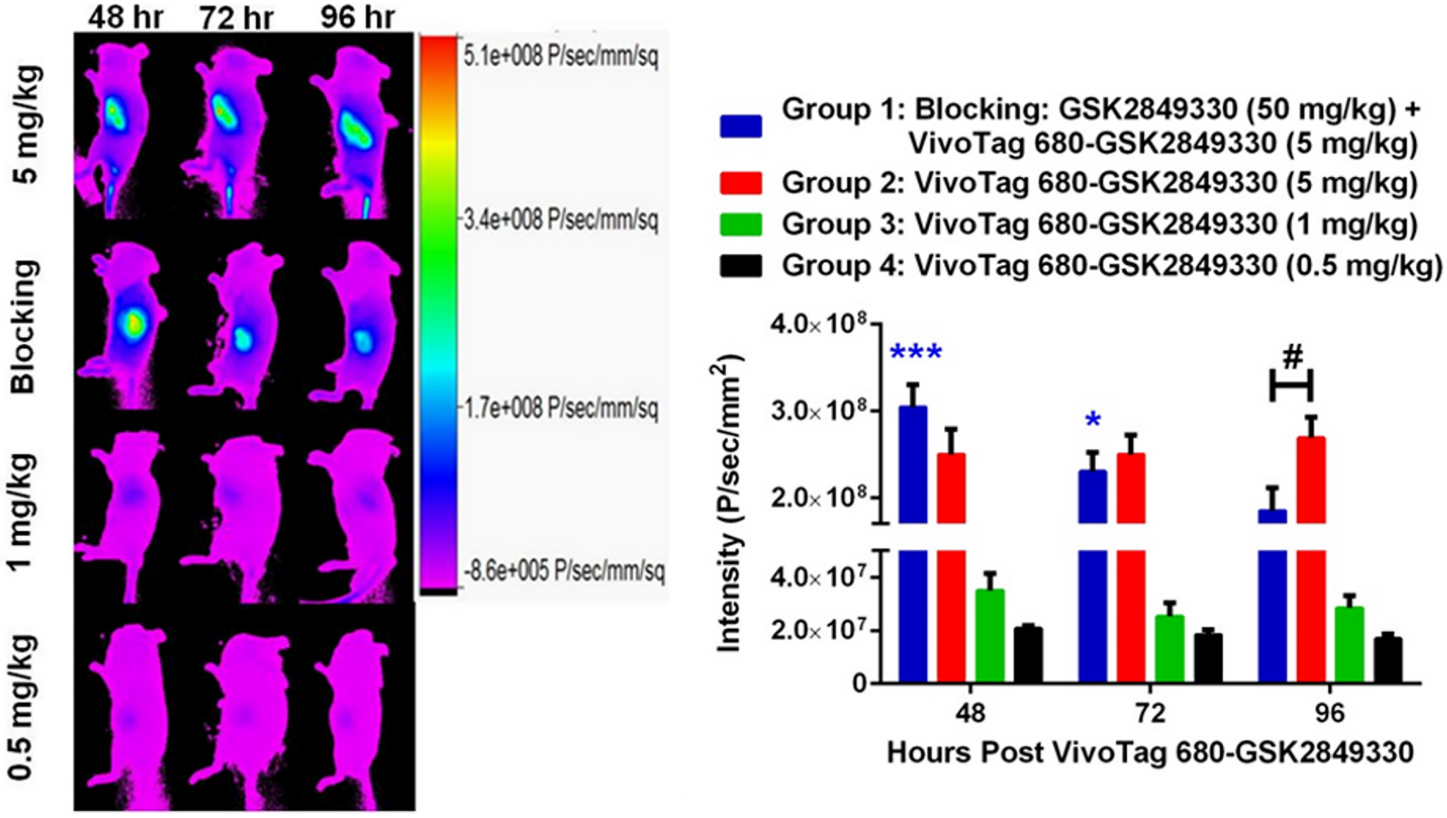
Tumor intensity at 48, 72, and 96 hours post injection of VivoTag 680-GSK2849330 in BxPC3 xenograft tumor bearing mice as measured in-vivo using optical imaging. Group 1 (blocking) received 5 mg/kg of VivoTag 680-GSK28493330 24 hours post injection of GSK2849330 (50 mg/kg). Groups 2, 3, and 4 received a single dose of VivoTag 680-GSK28493330 (5, 1, and 0.5 mg/kg, respectively). Groups 1 (blocking) and 2 (5 mg/kg) were significantly different compared to groups 3 (1 mg/kg) and 4 (0.5 mg/kg) at all time points. Group 1 (blocking) intensity decreased significantly over time: *p<0.05: 48 vs 96 hours, ***p<0.001: 48 vs 72 and 96 hours using 2-way ANOVA. Group 2 (5 mg/kg) showed significantly higher intensity compared to group 1 (blocking) at 96 hours: #p<0.05 using 1-way ANOVA. Data is presented as mean±SEM (S3 Table).

Optical imaging in mice with BxPC3 xenograft tumors was performed at 48, 72, and 96 hours post injection of the VivoTag 680-GSK2849330 (Fig 4). There were significant intensity differences between groups 1 (blocking), and 2 (5 mg/kg) compared with the lower dose groups 3 (1 mg/kg) and 4 (0.5 mg/kg) at all time points (p<0.001). The data showed no significant differences in optical intensities in the tumors between group 1 (blocking) and group 2 (5 mg/kg) at 48 and 72 hours. However, the tumor optical intensity was significantly decreased in group 1 (blocking) (1.85x10^8^±0.27x10^8^ P/sec/mm^2^) compared to group 2 (5 mg/kg) (2.69x10^8^±0.24x10^8^ P/sec/mm^2^, p<0.01) at 96 hours (Fig 4). Group 1 (blocking) showed a significant decrease over time in the tumor intensities (p<0.001: 48 hours vs 72 and 96 hours, p<0.05: 72 hours vs 96 hours) whereas group 2 (5 mg/kg) showed no similar trend over time.

### Ex-vivo confocal microscopy

**Fig 5:**
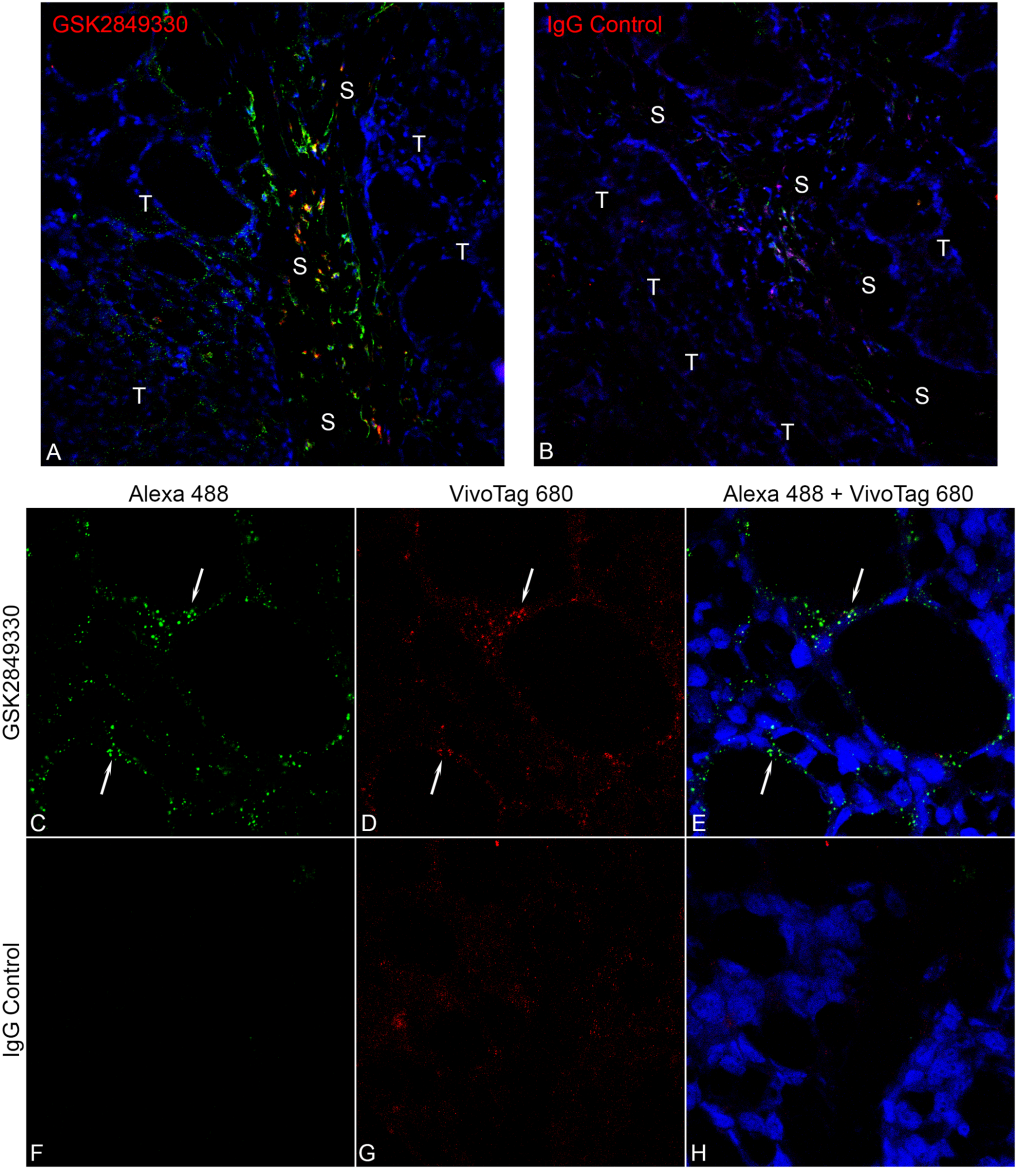
Confocal microscopy images of CHL-1 tumor samples collected 30 min post IV injection of either GSK2849330 (A, C, D, E) or IgG control (B, F, G, H) conjugated with VivoTag 680 (Red). The low magnification (20x) images show accumulation of the GSK2849330 (A) and the IgG control (B) labeled antibodies in the connective stromal tissue (S), but uptake of GSK2849330 into CHL-1 HER3 expressing tumor cells (T) was clearly distinguished. The high magnification images (63x) show GSK2849330 binding and internalization (arrows) in the individual tumor cell. Signal overlap between the Alexa 488 anti-human IgG secondary (green, C) and the directly labeled antibody (red, D) indicates specificity of the antibody localization on tumor cells (E). The CHL-1 tumor samples with IgG control showed little or no binding to tumor cells (F, G, H).

Confocal microscopy images (Fig 5) showed that the fluorescently labeled GSK2849330 was observed in CHL-1 tumors at the earliest time point (30 minute post-IVinjection) and was still evident in the tumor at 24 hours post administration. Signal patterns from the VivoTag 680 labeled antibody (red) and the Alexa 488 tagged anti-human IgG secondary (green) overlapped in nearly all cases. At low magnification (20x) accumulation within cells in the connective tissue in the stroma was observed (Fig 5A), but uptake into individual CHL-1 HER3 expressing tumor cells was clearly distinguished at high magnification (63x) as distinct punctate signal within the cytoplasm (arrows in Fig 5C, 5D and 5E). VivoTag 680 labeled IgG control did show some uptake by cells within the connective tissue stroma (Fig 5B) similar to that seen in the GSK2849330 treated animals, but to a much lesser extent. No specific CHL-1 tumor cell labeling was observed with the VivoTag 680 labeled IgG (Fig 5F, 5G and 5H).

### Magnetic resonance imaging (MRI)

**Fig 6:**
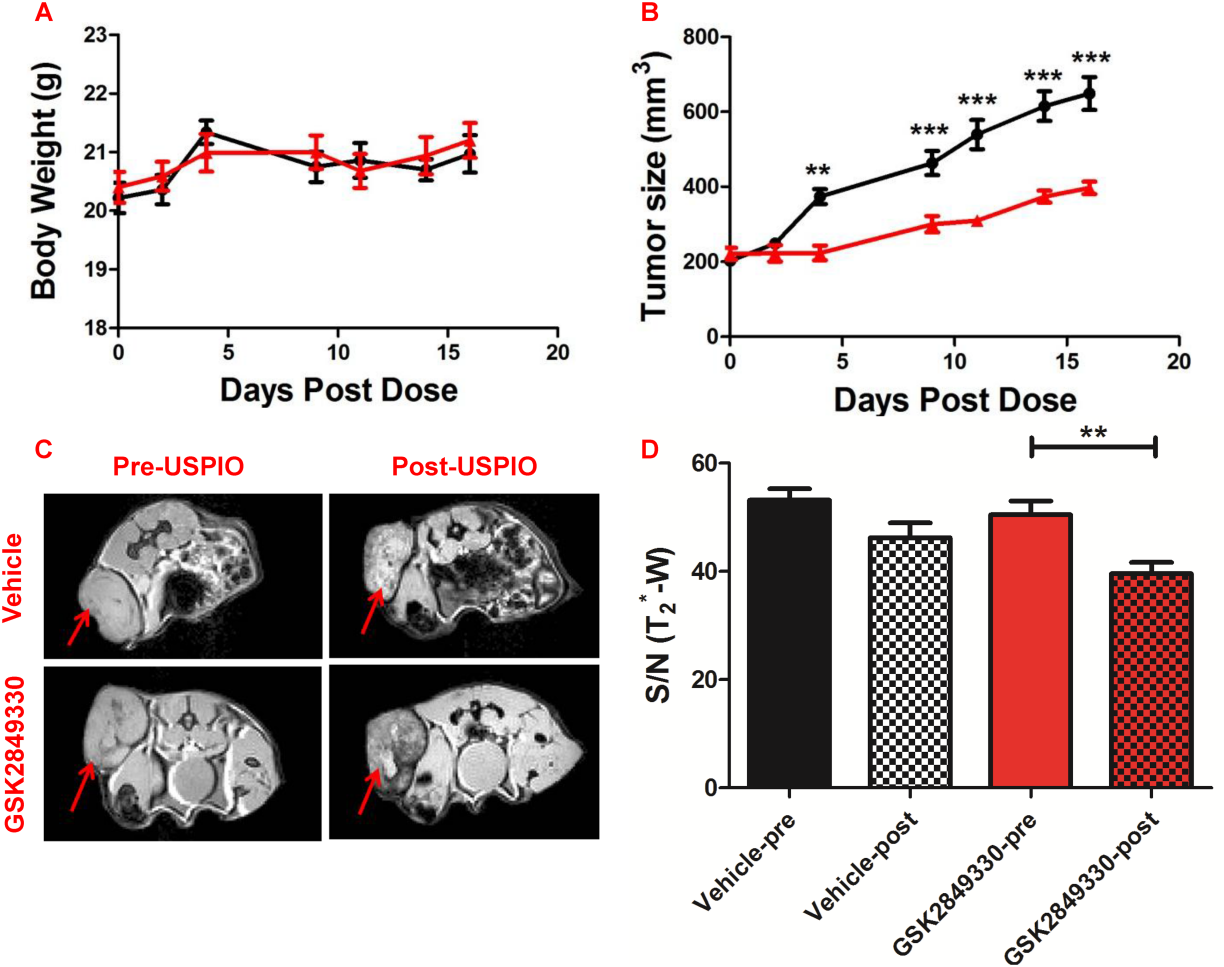
Experimental results from the USPIO MRI study. No difference in body weight (A) was observed between groups. Tumor growth (B) was significantly inhibited in the GSK2849330 treated group (red) compared to the vehicle group (black). **p<0.01, ***p<0.001 using 2 way ANOVA. Axial MRI slices (C) showing T_2_*-w images pre-USPIO and 24 hours post injection of ferumoxytol (Post-USPIO). Red arrows indicate xenograft tumor location. Tumor signal/noise ratio (S/N (T_2_*-w)) decreased significantly post-USPIO injection in the GSK2849330 treated group (red bars). However, no difference was found in the vehicle group (D). Data is presented as mean±SEM (S4 Table). **p<0.01: Unpaired t-test (two-tailed).

After 2 weeks of chronic treatment (3 times/week) with GSK2849330 in CHL-1 xenograft tumor-bearing mice, no mortality or differences in body weight were observed between groups (Fig 6A). Tumor size (Fig 6B) increased with time in the vehicle group; however, tumor growth was significantly reduced in the GSK2849330 treated group. Following administration of ferumoxytol (USPIO), one animal was excluded from the MRI analysis in the GSK2849330 treated group due to a high concentration of iron and very dark MR signal in the tumor which made analysis impossible. MR images (Fig 6C) showed differences in tumor signal intensity post USPIO injection compared to baseline. MR images (Fig 6C) showed differences in tumor signal intensity post USPIO injection compared to baseline. Signal/Noise (T_2_*-w) was decreased significantly post-USPIO injection compared to pre injection in the GSK2849330 treated group (Fig 6D) (50.5±2.5 vs 39.6±2.1; p<0.01) indicating that USPIO uptake was higher in the GSK2849330 treated group compared to the vehicle group.

**Fig 7:**
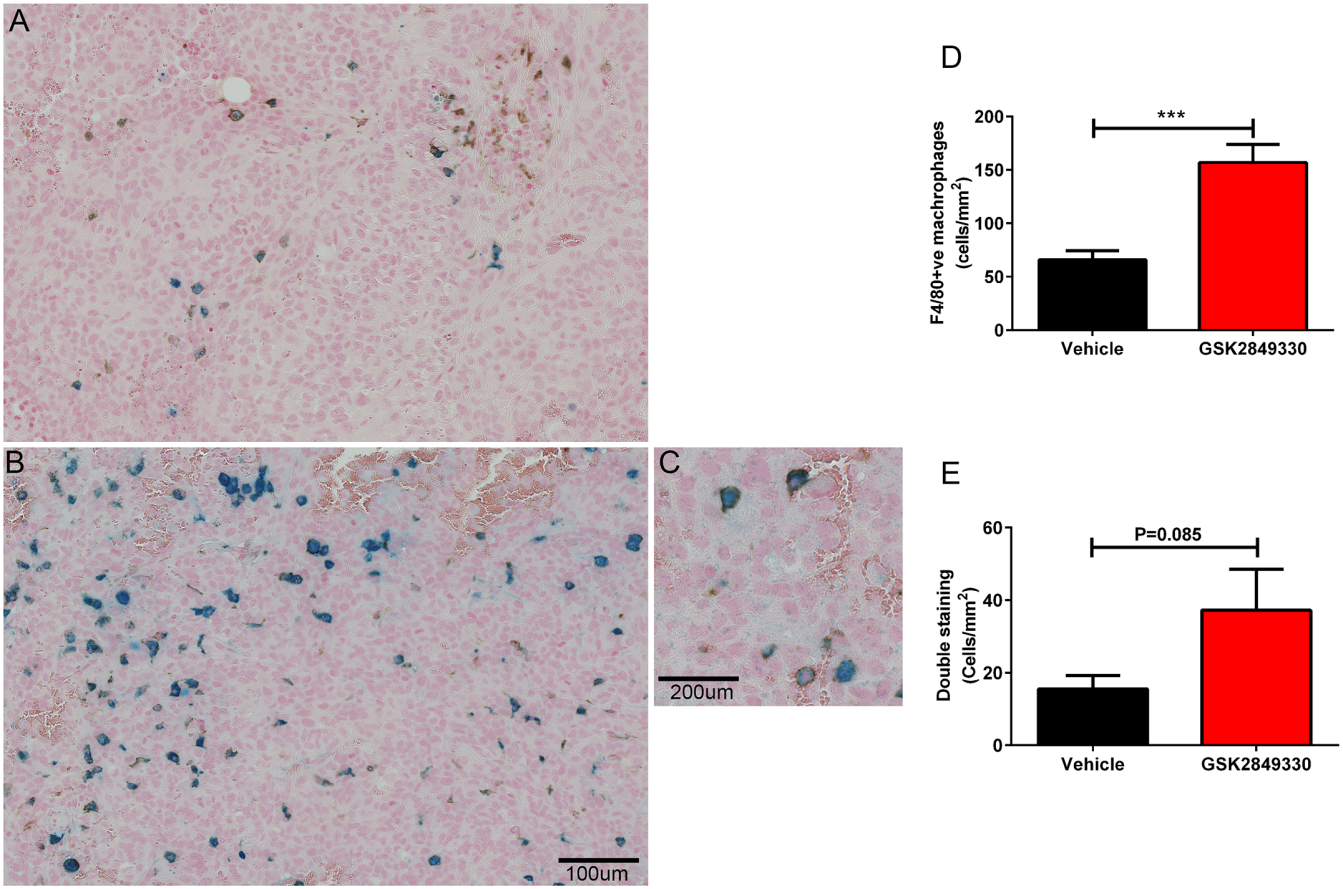
Immunohistochemistry analysis of tumors from the USPIO MRI study. Panels A and B show Prussian blue and F4/80 IHC staining for vehicle and GSK2849330 groups respectively. A region of tumor reflecting cytoplasmic iron staining coregistered with a sub population of plasma membrane F4/80 positive (F4/80+ve) cells is observed in C. The quantitative data (cells/mm^2^) reflects an increase in number of F4/80+ve macrophages (D) and a trend toward increased coregistered F4/80+ve macrophages with USPIO (E) in the GSK2849330 treated group compared to the vehicle group. Data is presented as mean±SEM (S5 Table). ***P<0.01: unpaired t-test (two-tailed).

Histological analysis of single sections of the tumors showed extensive necrosis in the vehicle group, but not in the GSK2849330 treated group. This may be a result of the increased tumor size in the vehicle group. Therefore, the quantitative analysis of USPIO and F4/80-positive (F4/80+ve) macrophages was performed separately for viable tumor and for cyst/necrotic like regions. The IHC staining confirmed the co-localization of ferumoxytol with a subset of F4/80+ve macrophages, and with little evidence of free iron (Fig 7A–7C).

The quantitative analysis of the viable tumor region showed a significant increase in F4/80+ve (cells/mm^2^) in the GSK2849330 treated group compared to the vehicle group (156.90±16.85 vs 65.98±8.31; respectively, P<0.001), Fig 7D. The dual staining in the same region showed a trend toward increased F4/80+ve—Perls double staining (cells/mm^2^) in the GSK2849330 treated group (37.24±11.34) vs the vehicle group (15.54±3.67) Fig 7E (p = 0.085).

However, a trend towards increased iron staining in the tumor’s viable region of the drug treated group vs vehicle as measured by perls staining alone was detected. Interestingly, the MRI data for the GSK2849330 treated group significantly correlated with the iron staining measured in the tumor’s viable region (r = -0.82, P<0.01). In addition, analysis of the cyst/necrotic like region of the tumor resulted in no differences between groups.

## Discussion

Herein we showed that Immuno-PET monitoring of a ^89^Zr-tagged monoclonal antibody directed against HER3 (^89^Zr-GSK2849330) can be used for non-invasive kinetic assessment of the mAb biodistribution in tumors and other organs. In addition, the studies showed that biodistribution of a single dose of ^89^Zr-GSK2849330 could be monitored non-invasively by PET imaging out to 144 hours. Furthermore, ^89^Zr-GSK2849330 uptake in tumor and the normalized tumor to blood ratios at 144 hours post injection of ^89^Zr-GSK2849330 were clearly differentiated between the HER3 expressing CHL-1 and non-expressing MIA-PaCa-2 xenograft tumors.

When blocking the HER3 receptor with a large non-labeled dose of GSK2849330 (50 mg/kg) administered 16 hours before injecting ^89^Zr-GSK2849330, liver uptake was substantially decreased while blood and CHL-1 xenograft tumor uptake was significantly increased up to 144 hours post injection of ^89^Zr-GSK2849330. Since the liver has a very low level of HER3 expression[24], the high uptake of ^89^Zr-GSK2849330 in the liver may be related to the binding of GSK2849330 to the FcγRs receptors since GSK2849330 is an anti-HER3 monoclonal antibody with optimized antibody dependent cell cytotoxicity (ADCC) and complement dependent cytotoxicity (CDC) activity. The gradual temporal increase of ^89^Zr-GSK2849330 uptake in CHL-1 tumor suggests a significant portion of the uptake was mediated by cellular uptake via internalization[25].

Results of the dose escalation study are consistent with a saturable hepatic uptake process and saturable (target mediated) drug disposition in tumor expressing HER3. All groups showed a high uptake of ^89^Zr-GSK2849330 in liver, and this uptake decreased significantly when an increasing dose of non-labeled GSK2849330 was administered simultaneously with the ^89^Zr-GSK2849330 tracer dose. Reduced uptake of ^89^Zr-GSK2849330 in liver in the presence of excess unlabeled GSK2849330 was associated with increased concentrations of ^89^Zr-GSK2849330 in blood, which appeared to drive increased concentrations in other organs (which presumably do not possess the saturable uptake process present in liver). In HER3-expressing tumors, the decreased tumor to blood ratio that was observed when ^89^Zr-GSK2849330 was co-injected with an increasing dose of the non-labeled GSK2849330 was presumably a result of in-vivo competition for the HER3 binding sites.

There have been a number of Immuno-PET preclinical biodistribution studies performed to evaluate antibodies targeting HER3 in human xenograft tumor-bearing mice[26–28]. These studies were performed in different tumor cell-lines which present with different tumor biology including HER3 expression level, making it difficult to compare across studies. Interestingly, the uptake of ^89^Zr-GSK2849330 in the liver was higher than previously reported for other HER3 targeting mAbs, and it exhibited a marked dose dependent uptake suggesting a saturable binding uptake process. This may be a consequence of the Fc-engineering which has been optimized in GSK2849330 to enhance FcγRs interactions and complement activation. Of note, RG7116 is an ADCC enhanced antibody [27] which has shown some dose dependence in the liver, but not to the same degree as observed with GSK2849330. However, GSK2849330 showed similar [27] to higher [28] tumor to blood uptake ratio in HER3 positive expressing xenograft tumors and comparable ratio in HER3 negative expressing xenograft tumors at 144 hours post injection of ^89^Zr-GSK2849330. These data suggest similar specificity for the various HER3 antibodies in the different human cell derived xenograft tumors. Notably, the uptake of ^89^Zr-GSK2849330 was similar in all other organs compared to the other antibodies targeting HER3 with the exception of much higher uptake of ^89^Zr-RG7116 [27] in the spleen compared to ^89^Zr-GSK2849330.

The optical imaging study was performed in BxPC3 human pancreatic xenograft mouse model as a second HER3 positive xenograft tumor. Again, the data showed a dose dependence of GSK2849330 in tumor, and the uptake of the fluorescently labeled GSK2849330 was significantly greater in the high dose group. This finding confirmed the results from the previous biodistribution study using ^89^Zr-GSK2849330. The blocking dose (50 mg/kg) of the non-labeled GSK2849330 given 24 hours prior to the fluorescently labeled GSK2849330 significantly decreased the tumor uptake at 96 hours when compared to the non blocking group. However, the effect of the blocking dose was observable in the ^89^Zr-GSK2849330 experiment only after normalizing the tumor to blood ratio. This difference in observation between experiments could be related to the difference in HER3 level expression, and the vascular density in the CHL-1 versus the BxPC3 xenograft tumor[29, 30]. Additionally, there may be differences in residualization and/or internalization properties between the VivoTag 680 optical and the Zirconium-89 nuclear tags used to label GSK2849330[25, 30, 31].

Our observations at the cellular level showed that tumor and tissue uptake was rapid: fluorescently labeled GSK2849330 was detected by confocal microscopy in the cytoplasm of CHL-1 tumor cells within 30 minutes of injection. High accumulation in particular cells within the connective stromal tissue was observed. A similar pattern, but with lower signal intensity was observed in animals treated with labeled IgG control antibody. However, GSK2849330 showed a specific cytoplasmic punctate staining pattern within individual CHL-1 cells, while the IgG treated animals did not. As the localization of both the IgG and the GSK2849330 was observed within the cells of the stroma, it may suggest that the uptake by these cells was most likely due to FcγRs interaction, and further evaluation would be warranted to determine this cellular composition. The distinct punctate cytoplasmic localization observed within the tumor cells closely resembled that observed in in-vitro studies (data not shown) using cultured CHL-1 cells incubated with labeled GSK2849330 which was internalized and co-localized within endosomes after only 15 minutes, suggesting that this also is a result of antibody binding of the HER3 receptor and subsequent internalization. Time points out to and including 24 hours post injection showed similar results. Notably at early time points (30 minutes and 1 hour), specific labeling of CHL-1 cells tended to be localized to those cells in close proximity to the stroma and associated vasculature as in many instances cells located a greater distance from these vessels had little or no visible label.

The biodistribution experiments of GSK2849330 showed that the uptake in tumor is affected not only by the HER3 expression level at the tumor but also by the complexity of the biological system which includes the expression level of the HER3 and FcγRs receptors in other organs, the vasculature density in the tumor lesions which may affect the transport, the clearance of the monoclonal antibody, and the dose and the timing of the treatment. The pharmacodynamics data showed that short term dosing of GSK2849330 significantly inhibited tumor growth in the CHL-1 human xenograft tumor mouse model. The MRI data showed a significant decrease in tumor signal intensity post USPIO (ferumoxytol) injection in the GSK2849330 treated animals compared with baseline which was associated with a significant increase of tumor F4/80+ve macrophage recruitment compared to the vehicle group as quantitatively measured by immunohistochemistry. These results suggest that ferumoxytol contrast enhanced MRI can be used to detect macrophage recruitment in tumor xenografts and monitor the response to antibodies such as GSK2849330 which are expected to affect macrophage function (ADCC) and/or recruitment to tumor. The increased macrophage recruitment in the tumor is most likely a result of the macrophage phagocytic activity as a secondary mechanism of action for ADCC enhanced GSK2849330 which was shown with other therapeutic antibodies[32, 33]. We described above the potential FcγRs interaction with GSK2849330 in liver cells as a dose dependent effect of GSK2849330 in the liver, and in cells within the connective stromal tissue of the tumor. However, further investigation into the role of FcγRs in increased macrophage recruitment with GSK2849330 in addition to the main action of inhibition of HER3 mediated cell signaling is warranted.

## Clinical translational

A phase 1 clinical trial (ClinicalTrials.gov Identifier: NCT02345174) of GSK2849330 has been initiated to characterize the biodistribution and dose-receptor occupancy relationship of GSK2849330 in patients with advanced HER3 expressing solid tumors using Immuno-PET imaging. By combining molecular imaging approaches (e.g. optical & nuclear) GSK2849330 biodistribution and dose response studies can be used to help guide optimal dose and dosing paradigm selection for future clinical development. Furthermore, ^89^Zr-GSK2849330 could be used diagnostically to differentiate tumor lesions expressing HER3 and monitor the response to treatment in patients with metastatic cancer by measuring the drug uptake in the different tumor lesions thereby addressing variability between patients, within patients and within lesions. Ferumoxytol, which is an FDA approved agent for the treatment of iron deficiency in adult patients, has been used as an MRI contrast agent for macrophage assessment. The ferumoxytol-enhanced MRI work presented here illustrates that the immune system antitumor response to GSK2849330 can be monitored, potentially providing a clinically translatable technique to non-invasively evaluate macrophage recruitment to tumor and serve as a surrogate marker for ADCC activity in patients. In conclusion, these imaging approaches presented in this work potentially provide clinically translatable, non-invasive techniques to support dose optimization, and assess aspects of immune activation and anti-tumor responses.

## Supporting information

S1 Table Fig 2 data. Immuno-PET study: Tumor uptake data (Fig 2A data), Blood uptake data (Fig 2B data) and the normalized tumor to blood ratio data (Fig 2C data).
(PDF)

S2 Table Fig 3 data. Dose escalation study: ex-vivo biodistribution data (Fig 3A and 3B data), normalized liver uptake to blood uptake ratio data (Fig 3C data), and normalized tumor uptake to blood uptake ratio data (Fig 3D data).
(PDF)

S3 Table Fig 4 data. Optical imaging study: Tumor optical intensity data (Fig 4 data).
(PDF)

S4 Table Fig 6 data. Experimental results from the USPIO MRI study: Body weight data (Fig 6A data), Tumor growth data (Fig 6B data), and tumor signal/noise ratio (S/N (T_2_*-w)) data (Fig 6D data).
(PDF)

S5 Table Fig 7 data. Immunohistochemistry analysis of tumors from the USPIO MRI study: Quantitative data for F4/80+ve macrophages (Fig 7D data), and quantitative data for the double staining for F4/80+ve—Perls (Fig 7E data).
(PDF)
