# Supplementary material for: Non invasive imaging assessment of the biodistribution of GSK2849330, an ADCC and CDC optimized anti HER3 mAb, and its role in tumor macrophage recruitment in human tumor-bearing mice
Source: PLoS One. 2017 Apr 27;12(4):e0176075. doi: 10.1371/journal.pone.0176075 (PMC5407619; doi:10.1371/journal.pone.0176075)
Supplement: S1 Table — Immuno-PET study: Tumor uptake data (Fig 2A data), Blood uptake data (Fig 2B data) and the normalized tumor to blood ratio data (Fig 2C data). (PDF) [file pone.0176075.s001.pdf]

S1 Table

Fig 2A data: Tumor uptake (%ID/g), n = 5 per group						
Hours post ^{89}Zr-GSK2849330 injection	Group 1 (MIA-PaCa-2): ^{89}Zr-GSK2849330 (0.5 mg/kg)		Group 2 (CHL-1): ^{89}Zr-GSK2849330 (0.5 mg/kg)		Group 3 (CHL-1): GSK2849330 (50 mg/kg) + ^{89}Zr-GSK2849330 (0.5 mg/kg)	
	Mean	SEM	Mean	SEM	Mean	SEM
**24**	4.08	0.39	3.68	0.54	4.40	0.20
**48**	3.08	0.21	3.52	0.37	4.72	0.25
**72**	2.42	0.12	3.26	0.46	5.02	0.24
**144**	1.70	0.11	2.54	0.37	5.18	0.20

Fig 2B data: Blood uptake (%ID/g) , n = 5 per group						
Hours post ^{89}Zr-GSK2849330 injection	Group 1 (MIA-PaCa-2): ^{89}Zr-GSK2849330 (0.5 mg/kg)		Group 2 (CHL-1): ^{89}Zr-GSK2849330 (0.5 mg/kg)		Group 3 (CHL-1): GSK2849330 (50 mg/kg) + ^{89}Zr-GSK2849330 (0.5 mg/kg)	
	Mean	SEM	Mean	SEM	Mean	SEM
**24**	2.73	0.17	2.60	0.28	8.40	0.41
**48**	0.94	0.10	1.06	0.13	6.26	0.25
**72**	0.89	0.08	1.05	0.07	4.88	0.22
**144**	0.75	0.08	0.66	0.10	2.92	0.19

Fig 2C data: Tumor to blood ratio, n = 5 per group						
Hours post ^{89}Zr-GSK2849330 injection	Group 1 (MIA-PaCa-2): ^{89}Zr-GSK2849330 (0.5 mg/kg)		Group 2 (CHL-1): ^{89}Zr-GSK2849330 (0.5 mg/kg)		Group 3 (CHL-1): GSK2849330 (50 mg/kg) + ^{89}Zr-GSK2849330 (0.5 mg/kg)	
	Mean	SEM	Mean	SEM	Mean	SEM
**24**	1.64	0.05	1.45	0.23	0.53	0.05
**48**	3.48	0.52	3.62	0.69	0.76	0.07
**72**	2.79	0.19	3.08	0.36	1.04	0.09
**144**	2.31	0.15	4.07	0.72	1.80	0.12
