# Supplementary material for: Non invasive imaging assessment of the biodistribution of GSK2849330, an ADCC and CDC optimized anti HER3 mAb, and its role in tumor macrophage recruitment in human tumor-bearing mice
Source: PLoS One. 2017 Apr 27;12(4):e0176075. doi: 10.1371/journal.pone.0176075 (PMC5407619; doi:10.1371/journal.pone.0176075)
Supplement: S2 Table — Dose escalation study: ex-vivo biodistribution data (Fig 3A and 3B data), normalized liver uptake to blood uptake ratio data (Fig 3C data), and normalized tumor uptake to blood uptake ratio data (Fig 3D data). (PDF) [file pone.0176075.s002.pdf]

S2 Table

Fig 3A & 3B data: Dose escalation study of ^{89}Zr-GSK2849330 (%ID/g), n = 4 per group										
	GSK2849330 (0 mg/kg)		GSK2849330 (0.3 mg/kg)		GSK2849330 (1 mg/kg)		GSK2849330 (3 mg/kg)		GSK2849330 (10 mg/kg)	
	Mean	SEM	Mean	SEM	Mean	SEM	Mean	SEM	Mean	SEM
**Blood**	0.46	0.03	0.57	0.04	1.05	0.09	2.28	0.25	8.43	0.61
**Lung**	1.04	0.10	1.85	0.17	2.71	0.22	3.30	0.37	6.65	0.62
**Heart**	1.01	0.01	0.90	0.10	1.22	0.10	1.59	0.18	3.76	0.24
**Pancreas**	0.48	0.03	0.85	0.34	1.36	0.16	1.35	0.14	1.77	0.14
**Tumor**	1.86	0.16	4.08	0.59	7.97	0.58	11.41	1.00	12.66	1.05
**Skin**	0.46	0.07	0.72	0.07	1.12	0.13	1.68	0.11	2.00	0.15
**Muscle**	0.33	0.04	0.56	0.06	0.53	0.04	0.83	0.11	1.42	0.11
**GI**	0.95	0.18	2.26	0.14	3.99	0.21	4.10	0.17	2.39	0.09
**Urine**	0.29	0.09	0.26	0.11	0.17	0.02	0.25	0.06	0.34	0.04
**Femur**	3.69	0.28	4.22	0.50	6.78	1.71	5.16	0.36	4.22	0.33
**Brain**	0.10	0.03	0.08	0.00	0.11	0.00	0.39	0.24	0.29	0.02
**Stomach**	0.42	0.07	0.77	0.05	1.65	0.17	1.32	0.38	1.52	0.20
**Thyroid**	0.90	0.09	2.42	0.55	3.63	0.27	4.23	0.08	3.65	0.35
**Liver**	27.77	1.85	15.17	1.05	12.64	1.17	10.30	0.63	8.96	0.35
**Kidneys**	10.96	1.33	9.29	0.94	12.04	0.74	11.26	0.85	12.67	1.20
**Spleen**	7.79	1.08	10.38	2.31	14.69	1.11	16.38	2.83	17.99	3.10

Fig 3C & 3D data: Dose escalation study of ^{89}Zr-GSK2849330, n = 4 per group										
	GSK2849330 (0 mg/kg)		GSK2849330 (0.3 mg/kg)		GSK2849330 (1 mg/kg)		GSK2849330 (3 mg/kg)		GSK2849330 (10 mg/kg)	
	Mean	SEM	Mean	SEM	Mean	SEM	Mean	SEM	Mean	SEM
**Liver/Blood**	60.46	4.12	27.22	3.19	12.22	1.19	4.64	0.43	1.07	0.03
**Tumor/Blood**	4.07	0.44	7.31	1.30	7.89	1.22	5.15	0.62	1.50	0.04
