# Supplementary material for: Non invasive imaging assessment of the biodistribution of GSK2849330, an ADCC and CDC optimized anti HER3 mAb, and its role in tumor macrophage recruitment in human tumor-bearing mice
Source: PLoS One. 2017 Apr 27;12(4):e0176075. doi: 10.1371/journal.pone.0176075 (PMC5407619; doi:10.1371/journal.pone.0176075)
Supplement: S3 Table — Optical imaging study: Tumor optical intensity data (Fig 4 data). (PDF) [file pone.0176075.s003.pdf]

**S3 Table**

| Fig 4 data: Tumor optical intensity (P/sec/$mm^2$), n = 4 per group | | | | | | | | |
|---|---|---|---|---|---|---|---|---|
| **Hours post $^{89}Zr$-GSK2849330 injection** | **Group 1: Blocking: GSK2849330 (50 mg/kg) + VivoTag 680-GSK2849330 (5 mg/kg)** | | **Group 2: VivoTag 680-GSK2849330 (5 mg/kg)** | | **Group 3: VivoTag 680-GSK2849330 (1 mg/kg)** | | **Group 4: VivoTag 680-GSK2849330 (0.5 mg/kg)** | |
| | **Mean** | **SEM** | **Mean** | **SEM** | **Mean** | **SEM** | **Mean** | **SEM** |
| **48** | 3.04E+08 | 2.60E+07 | 2.50E+08 | 2.93E+07 | 3.51E+07 | 6.34E+06 | 2.08E+07 | 1.07E+06 |
| **72** | 2.30E+08 | 2.20E+07 | 2.50E+08 | 2.22E+07 | 2.52E+07 | 5.16E+06 | 1.84E+07 | 1.73E+06 |
| **96** | 1.85E+08 | 2.65E+07 | 2.69E+08 | 2.39E+07 | 2.84E+07 | 4.59E+06 | 1.70E+07 | 1.66E+06 |
