# Supplementary material for: Non invasive imaging assessment of the biodistribution of GSK2849330, an ADCC and CDC optimized anti HER3 mAb, and its role in tumor macrophage recruitment in human tumor-bearing mice
Source: PLoS One. 2017 Apr 27;12(4):e0176075. doi: 10.1371/journal.pone.0176075 (PMC5407619; doi:10.1371/journal.pone.0176075)
Supplement: S4 Table — Experimental results from the USPIO MRI study: Body weight data (Fig 6A data), Tumor growth data (Fig 6B data), and tumor signal/noise ratio (S/N (T2*-w)) data (Fig 6D data). (PDF) [file pone.0176075.s004.pdf]

S4 Table

Fig 6A data: Body weight (g), n = 10 per group				
**Days post treatment**	**Vehicle group**		**GSK2849330 treated group**	
	**Mean**	**SEM**	**Mean**	**SEM**
**0**	20.22	0.26	20.40	0.26
**2**	20.36	0.25	20.59	0.25
**4**	21.34	0.20	20.99	0.32
**9**	20.75	0.26	21.00	0.29
**11**	20.86	0.30	20.68	0.29
**14**	20.70	0.18	20.94	0.32
**16**	20.97	0.32	21.20	0.30

Fig 6B data: Tumor size (mm^3), n = 10 per group				
**Days post treatment**	**Vehicle group**		**GSK2849330 treated group**	
	**Mean**	**SEM**	**Mean**	**SEM**
**0**	202.27	13.45	221.03	16.12
**2**	248.26	9.82	222.04	21.98
**4**	373.74	19.84	223.19	19.76
**9**	463.32	32.42	299.97	21.93
**11**	539.39	39.18	309.89	13.08
**14**	615.13	39.73	373.66	15.89
**16**	649.01	43.78	396.70	16.70

Fig 6D data: Tumor signal/noise ratio (S/N (T_2*-w)), n = 10 per group					
**Mouse number**	**Vehicle-pre**	**Vehicle-post**	**Mouse number**	**GSK2849330-pre**	**GSK2849330-post**
1	53.52	31.02	13	47.60	36.27
36	58.80	53.65	29	60.83	44.92
62	58.71	49.88	32	58.07	41.34
65	58.39	36.62	38	54.91	42.80
71	54.47	49.72	53	50.32	*
72	59.86	41.84	78	56.55	39.40
77	41.60	50.98	79	49.27	45.04
82	53.27	57.29	93	47.41	38.38
100	51.20	53.38	95	39.56	24.72
102	42.07	38.02	97	40.05	43.29
**Mean**	53.19	46.24	**Mean**	50.47	39.57
**SEM**	2.101	2.762	**SEM**	2.551	2.104
* Mouse 53 was excluded from the MRI analysis in the GSK2849330 treated group due to a high concentration of iron and very dark MR signal in the tumor which made analysis impossible.					
