# Supplementary material for: Non invasive imaging assessment of the biodistribution of GSK2849330, an ADCC and CDC optimized anti HER3 mAb, and its role in tumor macrophage recruitment in human tumor-bearing mice
Source: PLoS One. 2017 Apr 27;12(4):e0176075. doi: 10.1371/journal.pone.0176075 (PMC5407619; doi:10.1371/journal.pone.0176075)
Supplement: S5 Table — Immunohistochemistry analysis of tumors from the USPIO MRI study: Quantitative data for F4/80+ve macrophages (Fig 7D data), and quantitative data for the double staining for F4/80+ve—Perls (Fig 7E data). (PDF) [file pone.0176075.s005.pdf]

**S5 Table**

| Fig 7D & 7E data: Immunohistochemistry analysis of tumors, n = 10 per group | | | | | |
|---|---|---|---|---|---|
| **Vehicle group** | | | **GSK2849330 treated group** | | |
| **Mouse number** | **F4/80+ve machrophages (cells/mm$^2$)** | **Double staining (Cells/mm$^2$)** | **Mouse number** | **F4/80+ve machrophages (cells/mm$^2$)** | **Double staining (Cells/mm$^2$)** |
| **1** | 44.88 | 15.61 | **13** | 181.46 | 31.23 |
| **36** | 53.84 | 5.66 | **29** | 99.76 | 16.55 |
| **62** | 33.73 | 4.90 | **32** | 111.66 | 15.35 |
| **65** | 83.43 | 18.20 | **38** | 186.77 | 23.62 |
| **71** | 64.71 | 15.52 | **53** | 175.95 | 131.36 |
| **72** | 45.31 | 7.49 | **78** | 101.56 | 14.13 |
| **77** | 65.69 | 11.34 | **79** | 139.71 | 13.03 |
| **82** | 73.02 | 18.88 | **93** | 267.30 | 46.43 |
| **100** | 67.58 | 12.48 | **95** | 190.04 | 53.95 |
| **102** | 127.61 | 45.34 | **97** | 115.09 | 26.75 |
| **Mean** | 65.98 | 15.54 | **Mean** | 156.93 | 37.24 |
| **SEM** | 8.31 | 3.67 | **SEM** | 16.85 | 11.34 |
